# Expression of Foxtail Millet *bZIP* Transcription Factor *SibZIP67* Enhances Drought Tolerance in *Arabidopsis*

**DOI:** 10.3390/biom14080958

**Published:** 2024-08-07

**Authors:** Xinfeng Jia, Hanchi Gao, Lingxin Zhang, Wei Tang, Guo Wei, Juan Sun, Wangdan Xiong

**Affiliations:** 1 Grassland Agri-Husbandry Research Center, College of Grassland Science, Qingdao Agricultural University, Qingdao 266109, China; jiaxf@stu.qau.edu.cn (X.J.); 20212103047@stu.qau.edu.cn (H.G.); zlxin@stu.qau.edu.cn (L.Z.); tangwei@qau.edu.cn (W.T.); 2 Key Laboratory of National Forestry and Grassland Administration on Grassland Resources and Ecology in the Yellow River Delta, Qingdao Agricultural University, Qingdao 266109, China; 3 Qingdao Key Laboratory of Specialty Plant Germplasm Innovation and Utilization in Saline Soils of Coastal Beach, Qingdao Agricultural University, Qingdao 266109, China; 4 College of Horticulture and Landscape Architecture, Yangzhou University, Yangzhou 225009, China; gwei@yzu.edu.cn

**Keywords:** *bZIP*, drought, *Setaria italica*, transcription factor

## Abstract

Foxtail millet is a drought-tolerant cereal and forage crop. The basic leucine zipper (*bZIP*) gene family plays important roles in regulating plant development and responding to stresses. However, the roles of *bZIP* genes in foxtail millet remain largely uninvestigated. In this study, 92 members of the *bZIP* transcription factors were identified in foxtail millet and clustered into ten clades. The expression levels of four *SibZIP* genes (*SibZIP11*, *SibZIP12*, *SibZIP41*, and *SibZIP67*) were significantly induced after PEG treatment, and *SibZIP67* was chosen for further analysis. The studies showed that ectopic overexpression of *SibZIP67* in *Arabidopsis* enhanced the plant drought tolerance. Detached leaves of *SibZIP67* overexpressing plants had lower leaf water loss rates than those of wild-type plants. *SibZIP67* overexpressing plants improved survival rates under drought conditions compared to wild-type plants. Additionally, overexpressing *SibZIP67* in plants displayed reduced malondialdehyde (MDA) levels and enhanced activities of antioxidant enzymes, including catalase (CAT), superoxide dismutase (SOD), and peroxidase (POD) under drought stress. Furthermore, the drought-related genes, such as *AtRD29A*, *AtRD22*, *AtNCED3*, *AtABF3*, *AtABI1*, and *AtABI5*, were found to be regulated in *SibZIP67* transgenic plants than in wild-type *Arabidopsis* under drought conditions. These data suggested that *SibZIP67* conferred drought tolerance in transgenic *Arabidopsis* by regulating antioxidant enzyme activities and the expression of stress-related genes. The study reveals that *SibZIP67* plays a beneficial role in drought response in plants, offering a valuable genetic resource for agricultural improvement in arid environments.

## 1. Introduction

Plants often encounter various stresses during their growth, such as drought, salinity, and nutrient deficiency [1]. Drought is one of the major abiotic stresses, inhibiting plant growth and development and reducing crop yields and biomass production [2]. Plants have evolved complex signaling pathways to adapt to or avoid adverse environments, which are usually composed of stress receptors, signal transducers, metabolites, and the expression of specific stress-related genes [3,4]. Uncovering the mechanisms of drought tolerance and exploiting genes that can improve plant drought tolerance would be of significant value for crop breeding.

It has been reported that numerous transcription factors are involved in regulating stress tolerance to various abiotic stresses in plants [5]. Transcription factors can bind to the promoters of downstream genes and regulate their expression, thus enabling the plant to respond to abiotic stresses [6]. Plants can adapt to drought stress through abscisic acid-dependent (ABA-dependent) and ABA-independent pathways [7,8]. The accumulation of the plant phytohormone ABA can protect plants from drought stress [7,8]. Part of the ABA inducible downstream genes contain *cis*-elements in their promoters, and transcription factors can bind to promoters of these genes, thus enhancing plant drought tolerance [7,8].

Transcription factors, including the basic leucine zipper (*bZIP*), are essential components of plant signaling pathways, playing indispensable roles in plant growth and responding to abiotic stresses [9,10]. The bZIP transcription factors are among the most widely distributed and conserved proteins and have important regulatory functions in biotic and abiotic stresses [11,12]. The *bZIP* transcription factors are characterized by a basic region and a leucine zipper domain. The sequence of the basic region is relatively conserved, consisting of approximately 20 amino acid residues [11]. The *bZIP* transcription factors can bind to DNA as homologous or heterologous dimers and then inhibit or activate downstream genes [13]. Members of *bZIP* transcription factors have been identified in many plant species, such as *Arabidopsis*, rice, maize, sorghum, wheat, and switchgrass [14,15,16,17,18,19]. In *Arabidopsis*, 75 *bZIP* genes have been identified, and they are classified into ten subfamilies (named by groups A to I and S) based on the sequence homology [13]. In rice, there are 89 members of the *bZIP* genes [15]. Previous studies have shown that *bZIP* genes play important roles in the abiotic stress resistance of many plants [11,16,20,21]. Among the *AtbZIP* genes, 13 members in group A are responsible for abiotic stress and ABA signaling pathways [13]. When plants encounter and sense abiotic stresses or the endogenous level of ABA increases, transcription factors besides bZIP proteins are triggered and bind to the ABA-responsive elements (ABREs) of promoters in the ABA response genes [22]. Thus, ABA response genes trigger physiological reactions for drought resistance [22]. Similarly, overexpression of *OsbZIP23*, *OsbZIP45*, *OsbZIP66*, and *OsbZIP72* enhances rice drought tolerance and triggers the activation of ABA signaling pathways [22,23,24,25]. In wheat, *TabZIP60* and *TaABI5* improved plant freezing tolerance [16,26]. These findings demonstrate that the *bZIP* gene family is involved in response to abiotic stresses, functioning as a positive or negative regulator [27].

Foxtail millet is an important cereal and forage crop that can grow in semi-arid and arid areas [28,29]. The characterization of drought tolerance and sequenced genome make the foxtail millet a model plant for analyzing drought stress tolerance of the grass family [30]. Transcriptome and metabolome have been applied to analyze the biochemical responses under drought stress in foxtail millet [31,32,33,34]. Metabolic processes such as cutin and wax biosynthesis can facilitate the adaptation of foxtail millet to drought [32]. The analysis of the transcriptomic responses to drought stress in foxtail millet was conducted by comparing the reactions of drought-tolerant and drought-sensitive cultivars [35]. Among the differentially expressed genes, *bZIP* transcription factors were identified [35]. Additionally, drought response transcription factors in foxtail millet also included *bZIPs* during seed germination and early seedling stages in response to drought stress [32,36]. Despite these findings, an understanding of the molecular mechanisms that confer drought tolerance in foxtail millet and the specific roles of bZIP transcription factors within this context remains constrained.

Given that *bZIP* transcription factors have been demonstrated to play a critical role in drought responses, a better understanding of the functions of the *bZIP* family will bring new insights into drought tolerance in foxtail millet. In this study, we found that the expression pattern of *SibZIP67* was upregulated under drought stress conditions, suggesting it may be associated with drought tolerance. Consequently, *SibZIP67* was cloned, and ectopic was expressed in *Arabidopsis*. The drought stress tolerance of *SibZIP67* transgenic lines was measured, as well as water loss rate, malondialdehyde (MDA) content, and survival rates. The results of these analyses are presented and discussed in the context of the importance of *SibZIP67* in regulating stress responses and its potential value in crop and grass breeding.

## 2. Materials and Methods

### 2.1. Plant Material and Growth Conditions

Seeds of drought-tolerant foxtail millet variety (Jigu20) were germinated and cultured in Hoagland solution at 28 °C/16 h and 24 °C/8 h condition. Roots, stems, leaves of foxtail millet seedlings were sampled to analyze the RNA transcription at the six-leaf stage. Samples were collected and frozen in liquid nitrogen and stored at −80 °C for RNA transcription analysis.

*A. thaliana* Columbia-0 (WT) was grown in soil under greenhouse conditions at a temperature of 25 °C, with a 16 h/8 h photoperiod. Seeds of T3 homozygous transgenic lines were used for drought experiment analysis.

### 2.2. Transcriptome Analysis of Differential Expression Genes

The total RNA of leaves was extracted from stored samples using TRIzol reagent (Invitrogen, Waltham, MA, USA), and the high-quality total RNA (RIN ≥ 7) was used for transcriptome library construction. Transcriptome libraries were sequenced on the NovaSeq 6000 platform. Quality control of raw reads was performed to remove adapter sequences, and clean reads were mapped against the *Setaria italica* reference genome (*Setaria italica* v2.2, https://phytozome.jgi.doe.gov), accessed on 12 April 2021. The comparison of gene expression differences was carried out, and DEGs were obtained with a level of |log_2_FC| ≥ 1 and FDR < 0.05 [37].

### 2.3. RNA Extraction and Expression Analysis

Total RNA from the leaves and roots of foxtail millet was also extracted using TRIzol reagent (Invitrogen, CA, USA), and first-strand cDNA was synthesized using PrimerScript Reverse Transcriptase (Vazyme, Nanjing, China) according to the manufacturer’s instructions. The cDNA was used to validate RNA-seq results by RT-PCR and for gene cloning. Total RNA was also extracted from the *Arabidopsis* leaves of WT and transgenic lines, and the first-strand cDNA was synthesized for further gene expression analysis. qRT-PCR was carried out with SYBR Green (Takara, Tokyo, Japan) using the CFX96 (Bio-Rad Lab. Inc., Hercules, CA, USA). The *β-Actin* gene, *Seita.7G294000,* was used as the reference gene to normalize the target gene expression levels in green foxtail, and the 2^−ΔΔCT^ method was used to calculate the gene expression level. All qRT-PCR primers are shown in Supplementary Table S1.

### 2.4. Vector Construction and Plant Transformation

The full-length sequence of the *SibZIP67* gene was amplified from foxtail millet and subcloned into the modified vector pCAMBIA1301 (Supplementary Table S1) [38]. The final construct was confirmed by sequencing and introduced into *Agrobacterium* (strain GV3101). The Agrobacterium containing the constructs was used to transform into *Arabidopsis* with the floral dip method [39].

### 2.5. Phylogenetic Analysis

The bZIP transcription factors from rice and foxtail millet were downloaded from the Phytozome database (https://phytozome.jgi.doe.gov), accessed on 5 December 2023. Multiple sequence alignments were performed using Clustal X. The bZIP amino acid sequences were used to construct a phylogenetic tree with MEGA by the Neighbor-Joining method, and the bootstrap test was performed with 1000 iterations.

### 2.6. Stress Treatments and the Response of the Seeds to Mannitol and ABA

Plants of foxtail millet (Jigu 20) at the six-leaf stage were treated with 20% PEG-6000 for 2 h and 6 h, with the Hoagland solution containing no PEG as control. Roots and shoots of foxtail millet seedlings were sampled to analyze the gene expression responses at the six-leaf stage. Specifically, the 2-h PEG treatment was compared against a 2-h untreated control, and similarly, the 6-h PEG treatment was compared against a 6-h untreated control. Three replicates were performed.

WT and transgenic *Arabidopsis* seeds were harvested at the same time for seed germination analysis. Seeds were sterilized and sown on 1/2 MS medium containing 0, 200, 300 mM mannitol or 1 µM ABA. Seeds were vernalized at 4 °C for 2 days and grown at 24 °C under a 16 h/8 h photoperiod. The germination rate was counted every 12 h.

For osmotic stress treatment, seeds were germinated on 1/2 MS medium at 24 °C for 2 days and transferred onto MS medium plates supplemented with mannitol. The primary root length was measured seven days after the transfer [40].

To ascertain the in vitro water loss rate of the leaves, WT and transgenic *Arabidopsis* seeds were sown in pots containing a 3:1 mixture of sand and soil. The rosette leaves of 3-week-old seedlings were detached and their weight were promptly weighed [20]. The samples were then placed in a constant-humidity incubator set at a temperature of 25 °C, with their weights being meticulously monitored at one-hour intervals.

The formula for calculating the water loss rate is as follows:


Water loss rate (%) = (Initial weight-Final weight)/(Initial weight) × 100%

The “initial weight” is defined as the initial mass of the excised rosette leaves at the commencement of the experiment, while the “Final weight” denotes the mass of the leaves as recorded hourly. By persistently tracking and computing the weight discrepancies, one can ascertain the water loss rate of the samples at various time intervals.

For drought stress treatment, seeds were sown in pots containing a 3:1 mixture of sand and soil, and 3-week-old seedlings were left without water for 12 days and then watered. During the drought stress treatment, leaves were collected when the soil moisture was 30%. The freeze-dried and powered leaf samples were used to analyze gene transcription, the content of MDA, and enzyme assay activities of CAT, POD, and SOD [41]. The transcript levels of drought-related marker genes were examined via qRT-PCR, with primers listed in Supplementary Table S1. The content of MDA and enzyme assay activities of CAT, POD, and SOD were measured with enzyme kits (Keming Biotechnology, Suzhou, China) according to the manufacturer’s instructions. The survival rate is articulated as the percentage of individuals that demonstrate resilience in the face of desiccation, enduring a 12-day span without water before being rehydrated, which is quantified to reflect the number of survivors in proportion to the initial population size [20].

### 2.7. Statistical Analysis

The means and standard deviations were calculated. ANOVA was employed using IBM SPSS software (version 22), and differences were distinguished by the LSD test at the 0.05 probability level.

## 3. Results

### 3.1. Isolation and Classification Analysis of SibZIP Genes

**Figure 1:**
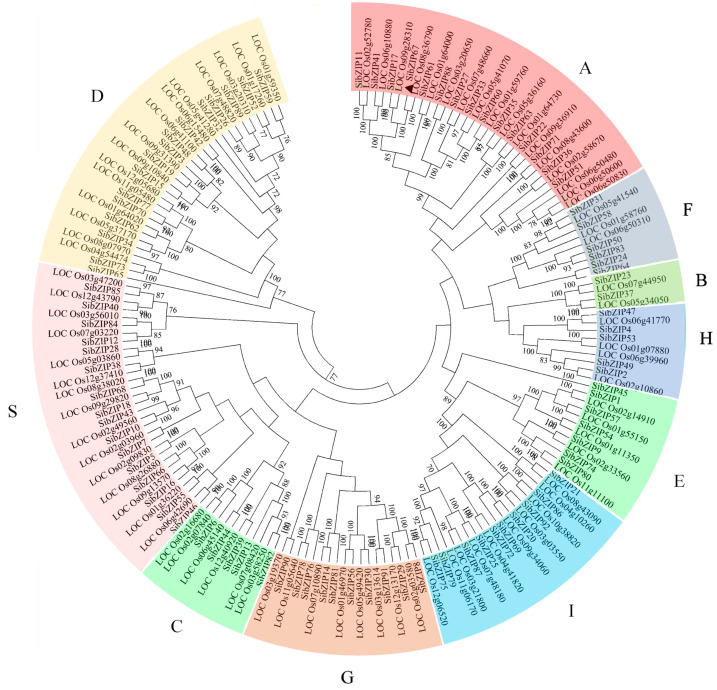
Phylogenetic analysis of bZIP proteins in foxtail millet and rice.

A total of 92 *SibZIP* genes were identified from the foxtail millet genome, and they were named according to their chromosomal location (Supplementary Table S2). The amino acid sequences of the *SibZIP* genes ranged in length from 140 to 759 residues. To analyze the evolutionary relationship among these genes, a phylogenetic tree was constructed using bZIP protein sequences from rice and foxtail millet (Figure 1). The bZIP members were also clustered into ten subfamilies.

### 3.2. Differentially Expressed Genes after PEG Treatment in Foxtail Millet

**Figure 2:**
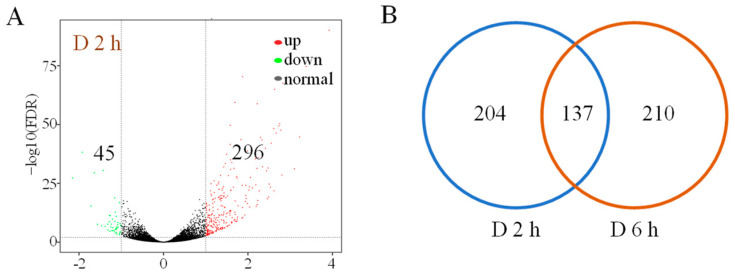

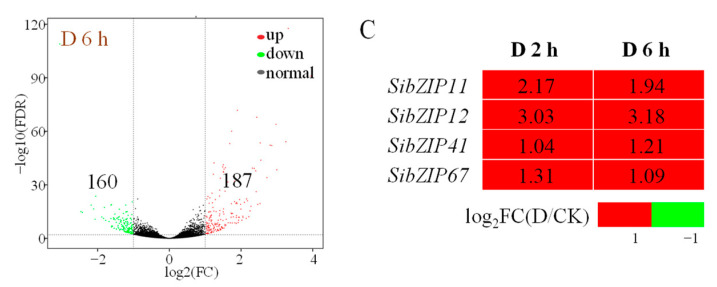
Differential expression genes in foxtail millet leave after PEG treatment. (**A**) Volcanic maps after PEG treatment. Green and red dots indicate down-regulated DEGs for log_2_(FC) ≤ −1 and up-regulated DEGs for log_2_(FC) ≥ 1, respectively. Black dots indicate no significant differences between transcriptomes. (**B**) Venn diagrams of co-regulated genes at 2 h and 6 h after PEG treatment. (**C**) Regulated *SibZIP* genes after drought treatment at both 2 h and 6 h. D refers to the expression level under PEG treatment, and CK refers to the control. The experiment contains three biological replicates.

RNA-seq analysis revealed 45 genes upregulated and 296 genes downregulated after 2 h of PEG treatment (Figure 2A, Table S3). After 6 h PEG treatment, 160 and 187 DEGs were up- or down-regulated, respectively (Figure 2A, Supplementary Table S3). Among the DEGs, 137 genes were co-regulated at both 2 h and 6 h, including four *bZIP* genes (*SibZIP11*, *SibZIP12*, *SibZIP41*, and *SibZIP67*) (Figure 2B,C). Gene *SibZIP11*, *SibZIP41*, and *SibZIP67* all belong to group A, and *SibZIP12* was in group S (Figure 1, Supplementary Table S4). Gene *SibZIP67*, closely clustered with the drought-related gene *OsbZIP66* (*LOC_Os08g36790*) [25], was selected for further investigation.

### 3.3. Bioinformatic and Expression Analysis of the SibZIP67 Gene

**Figure 3:**
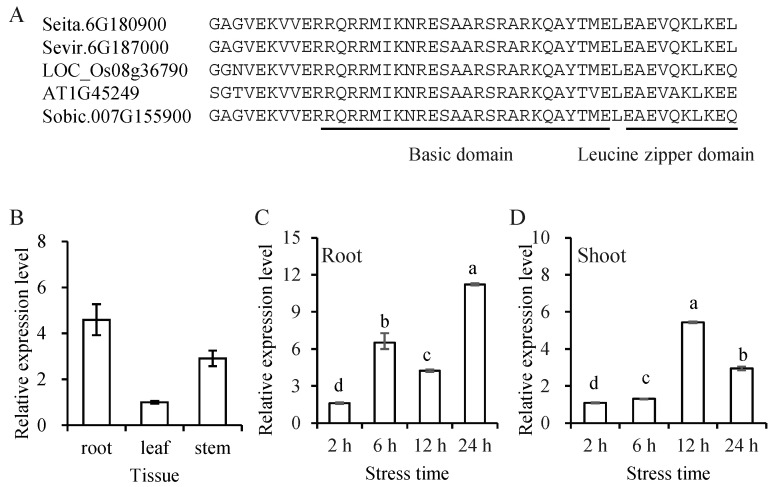
The conserved domain of SibZIP67 and expression pattern of *SibZIP67* in foxtail millet. (**A**) The conserved domain of SibZIP67 and its closely related sequences in rice, *Arabidopsis*, green foxtail, and sorghum. (**B**) Expression levels of *SibZIP67* in root, leaf, and stem under unstressed conditions. (**C**) The expression level of *SibZIP67* in roots after PEG treatment. (**D**) The expression level of *SibZIP67* in shoots after PEG treatment. The baseline expression of the *SibZIP67* gene at each time point in the control group was set to a value of 1. The relative expression levels of the *SibZIP67* gene in response to PEG treatment were ascertained by comparing the fold changes in gene expression between the treated samples and their corresponding controls at each respective time point. Lowercase letters indicate a significant difference at *p* < 0.05. Each experiment contains three biological replicates, and each with two technical replicates (means of n = 6 ± SD).

The coding sequence (CDS) of *SibZIP67* was 1083 bp in length, encoding 360 aa. Analysis of the deduced protein sequence revealed the presence of a basic DNA binding domain and a leucine zipper domain (Figure 3A). The expression level of the gene *SibZIP67* in the root at the six-leaf stage was found to be relatively higher than that in the stem and leaf (Figure 3B). The expression pattern of *SibZIP67* after PEG 6000 was examined using qRT-PCR. The expression level of *SibZIP67* was induced at 6 h and peaked at 24 h in root, and it was also induced and peaked at 12 h in shoot (Figure 3C,D). The results were consistent with the RNA-seq data, indicating that *SibZIP67* may be involved in foxtail millet drought stress response.

### 3.4. Analysis of Seed Germination and Growth with Exogenous ABA and Osmotic Treatments

**Figure 4:**
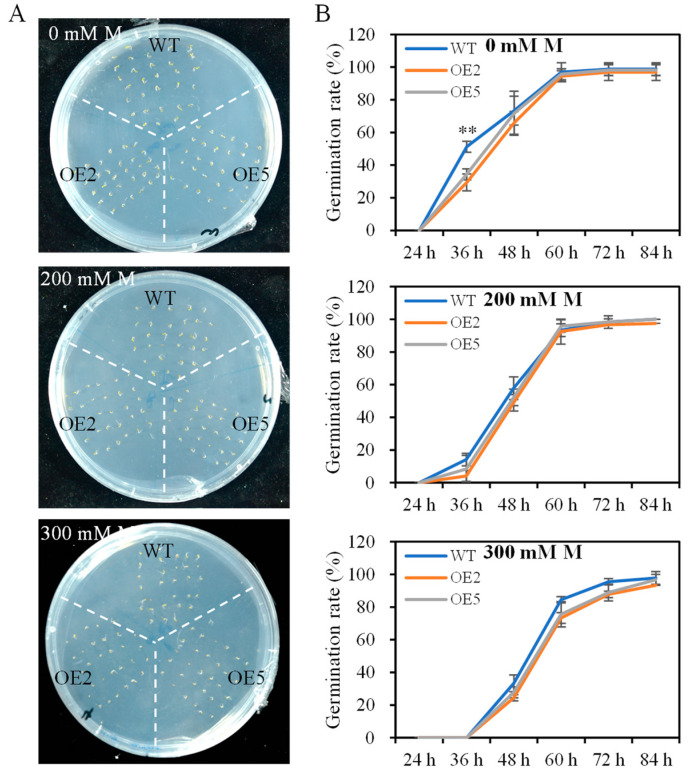
The germination rates of *SibZIP67* overexpressing lines and WT under mannitol treatment. (**A**) Comparison of germination of *SibZIP67* overexpressing lines and WT on 1/2 MS medium containing 0, 200, and 300 mM mannitol for 3 days. (**B**) Seed germination rates of *SibZIP67* overexpressing lines and WT on 1/2 MS medium containing 0, 200, and 300 mM mannitol. Data represent mean values ± SD from four biological replicates (n = 72). ** indicates *p* < 0.01.

*SibZIP67* was cloned and overexpressed under the control of the 35S-CaMV promoter to characterize its function in *Arabidopsis*. Seven overexpressing lines (OE1-7) were obtained, and two lines (OE2 and OE5) were selected for further germination and drought tolerance tests according to the *SibZIP67* expression level (Supplementary Figure S1). In the 1/2 MS medium, the germination rate of *SibZIP67* overexpressing lines at 36 h was lower than that of the wild-type (WT) *Arabidopsis* (Figure 4). The germination rates showed no apparent differences between *SibZIP67* transgenic lines and WT in 1/2 MS medium containing 200 or 300 mM mannitol (Figure 4). Additionally, the ABA sensitivity was also evaluated, and the seed germination rate of *SibZIP67* overexpressing lines was much lower than that of the WT, highlighting that *SibZIP67* overexpression in *Arabidopsis* increased the ABA sensitivity (Supplementary Figure S2).

**Figure 5:**
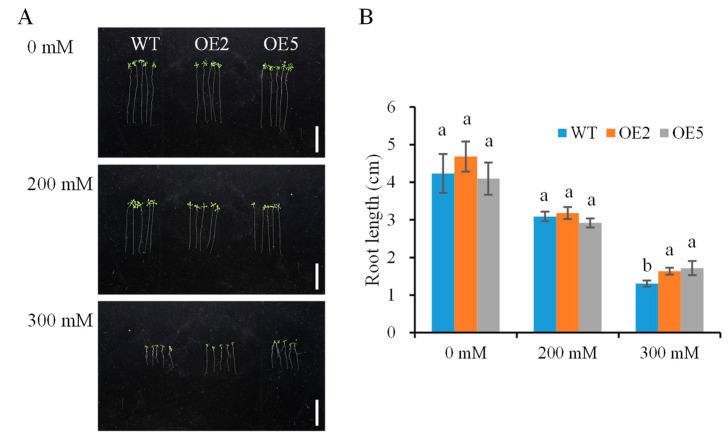
Phenotype and root length of *SibZIP67* overexpressing lines on a vertical plate under mannitol treatment. (**A**) Phenotypes of *SibZIP67* overexpressing lines and WT on 1/2 MS medium containing 0, 200, and 300 mM mannitol for 7 days. (**B**) Root length between transgenic and WT seedlings on 1/2 MS medium containing 0, 200, and 300 mM mannitol for 7 days. All data were analyzed for five biological replicates (n = 30). Lowercase letters indicate a significant difference at *p* < 0.05.

To assess the seedling response of *SibZIP67* transgenic lines to osmotic stress, the plants were subjected to 1/2 MS medium containing mannitol at the stage of post-germination growth (Figure 5). The root growth was reduced under osmotic stress for both transgenic lines and WT. However, no difference was observed in the main root length between transgenic lines and WT under normal conditions. Moreover, the roots of transgenic lines were longer than those of the WT plants under 300 mM mannitol treatment.

### 3.5. Analysis of Plants under Drought Stress

**Figure 6:**
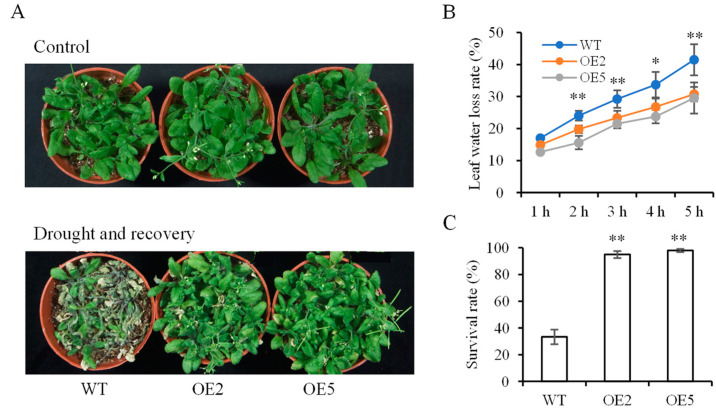

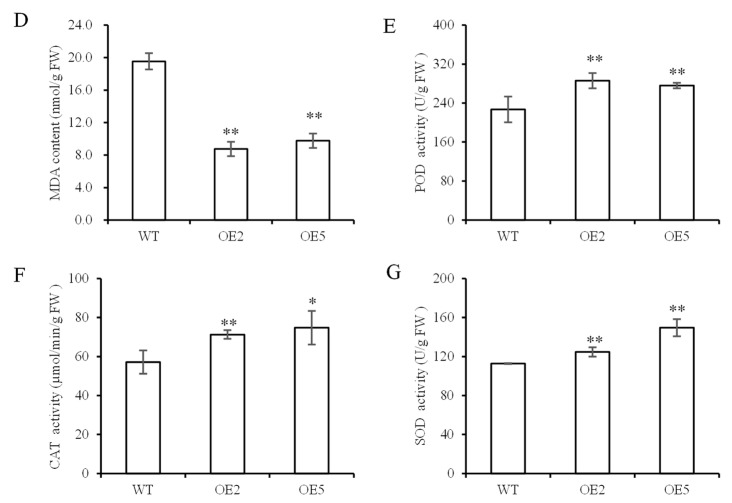
Drought tolerance of *SibZIP67* overexpressing lines. (**A**) Phenotype of *SibZIP67* overexpressing lines and WT after drought treatment. (**B**) Water loss rate of detached leaves. (**C**) Survival rate statistics after withholding water for 12 days and re-watering for 3 days. (**D**) MDA content in the leaves of transgenic and WT plants under drought stress. (**E**–**G**) Activities of POD (**E**), CAT (**F**), and SOD (**G**) activities of transgenic and WT plants under drought stress. Data in (**B**,**C**): means of n = 30 ± SD from three independent experiments. Data in (**D**–**G**): means of n = 6 ± SD from three independent experiments. * and ** indicate *p* < 0.01 and *p* < 0.01, respectively.

To assess the impact of *SibZIP67* transgenic lines on drought tolerance, the water loss rate of detached leaves was measured. The results demonstrated that the leaves of transgenic lines exhibited a relatively lower water loss rate (Figure 6B). Furthermore, the plants were withholding water. The MDA content was measured when the soil moisture content was 30%. The MDA content was higher in the WT plants than that in the transgenic lines (Figure 6D). The activities of the antioxidant enzymes superoxide dismutase (SOD), catalase (CAT), and peroxidase (POD) in WT were much lower than those in transgenic plants (Figure 6E,F). After withholding water for 12 days and rewatering for 3 days, the transgenic lines had higher survival rates than the WT plants (Figure 6C).

**Figure 7:**
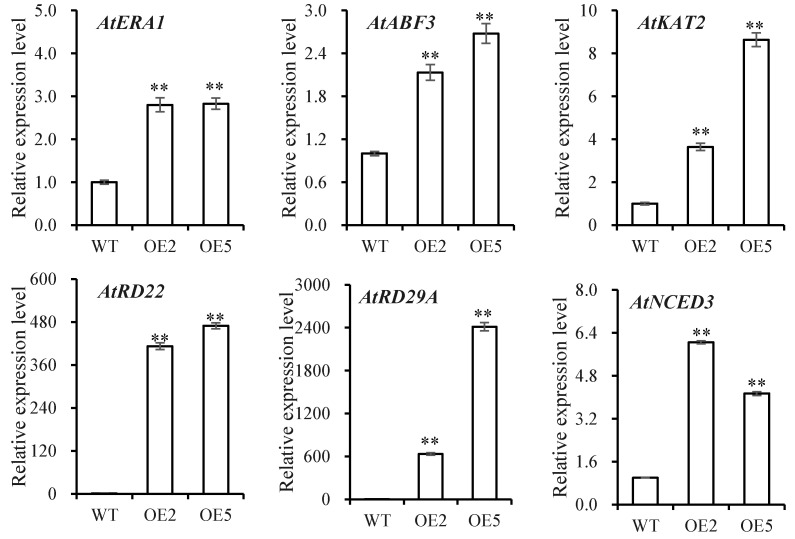

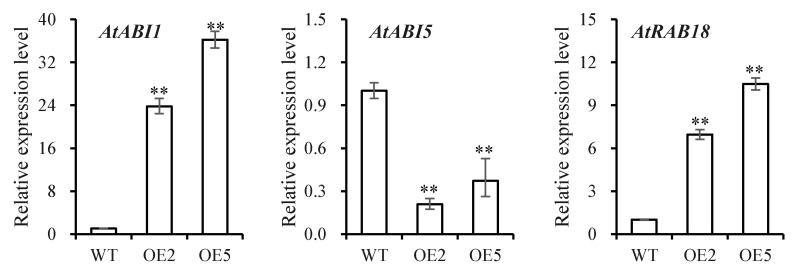
Transcript levels of stress-related marker genes in *SibZIP67* overexpressing lines and WT plant. Leaves were collected when the soil moisture content was 30%. Each experiment contains three biological replicates, and each with two technical replicates (means of n = 6 ± SD). ** indicates *p* < 0.01.

To further analyze the molecular mechanism underlying drought response, the expression levels of ABA-responsive and stress-responsive genes involved in the drought pathway were also analyzed. The expression of *AtRD29A*, *AtRD22*, *AtABF3, AtABI1, AtERA13, AtKAT2, AtRAB18,* and *AtNCED3* genes was significantly induced in overexpressing plants, while the expression of *AtABI5* was repressed (Figure 7).

## 4. Discussion

Foxtail millet is a multifunctional plant that can be used as a food and forage crop [28,29]. Drought represses crop growth and decreases their productivity. Many foxtail millet varieties have been cultivated and recognized for their ability to thrive in semi-arid and arid conditions, and studies have characterized and analyzed the drought resistance mechanism [42,43,44]. Transcription factors play pivotal roles in plant drought resistance [5,11,12]. The *bZIP* transcription factors constitute a conserved gene family, playing indispensable roles in plant growth, development, and stress responses [10]. Consequently, *bZIP* transcription factors were characterized from the foxtail millet genome, and *SibZIP* genes that exhibit responsiveness to drought conditions were singled out for subsequent analysis. A total of 92 *SibZIP* genes were identified in the foxtail millet genome. The *SibZIP* family is the same size as that of other plants like *Arabidopsis* (75), rice (89), and *Hordeum vulgare* (92) [13,15,45].

For previous studies, the treatment was set and analyzed between 6 h and 1 week [32,33,34,46]. Following a week of drought stress with the water content of the sand soil mixture at 12  ±  1%, 1367 and 2191 DEGs were annotated in the seedling leaves of drought-sensitive and drought-tolerant millet varieties, respectively [32]. Through RNA-seq analysis, 2393 and 3078 were DEGs expressed in foxtail millet leaves under light drought stress and heavy drought stress treatment [33]. In this study, the foxtail millet seedlings were treated with PEG for 2 h and 6 h, and trying to evaluate the early drought responses with transcriptome analysis. Here, only 137 genes were co-regulated after PEG treatment for 2 h and 6 h (Figure 2). This may be due to the short processing time or the different processing methods. Under drought stress, plants may respond by regulating the expression levels of more genes. Four out of 137 DEGs (*SibZIP11*, *SibZIP12*, *SibZIP41*, and *SibZIP67*) belong to the *bZIP* family. Transcription factors, besides *bZIP* genes, also respond under drought conditions during the early response of foxtail millet to water scarcity, indicating they may play important roles in protecting plants from drought stress [32]. Interestingly, *SibZIP67* was found to be rapidly upregulated following PEG treatment in this study and showed a similar response to prolonged drought stress in a previous study [32]. Therefore, the early response and high levels of upregulation in stressed leaves of foxtail millet make *SibZIP67* an interesting candidate deserving further characterization.

Further experiments confirmed that overexpression of *SibZIP67* enhanced the plant’s drought tolerance. Among the four regulated *SibZIP* genes, three regulated genes (*SibZIP11*, *SibZIP41*, and *SibZIP67*) were located in subfamily A, and *SibZIP12* belonged to subfamily S (Figure 1). Previous studies have revealed that *Arabidopsis bZIP* genes in group A (such as *AtbZIP36*, *AtbZIP37*, and *AtbZIP39*) participate in drought stress responses [13]. In rice, *OsbZIP23*, *OsbZIP46*, and *OsbZIP66,* belonging to group A, have been reported to be involved in drought stress [24,25,47]. Moreover, gene *SibZIP67* was closely clustered together with *OsbZIP66* (*LOC_Os08g36790*), which had been revealed to positively regulate drought response by regulating the expression of *OsLEA3* in rice [25]. There is an 82.9% similarity between SibZIP67 and its ortholog in rice OsbZIP66. *OsbZIP66* also rapidly induced under drought stress and enhanced the plant drought tolerance through the ABA-dependent pathway [48]. Thus, *OsbZIP66* was also named as the *transcription factor responsible for ABA regulation 1* (*TRAB1*) [48]. The close evolution relationship and similar function of these *bZIP* genes indicated that homologous genes have evolved to perform similar functions. Moreover, the results indicated that *SibZIP* genes would also play important roles in conferring drought tolerance to foxtail millet.

**Figure 8:**
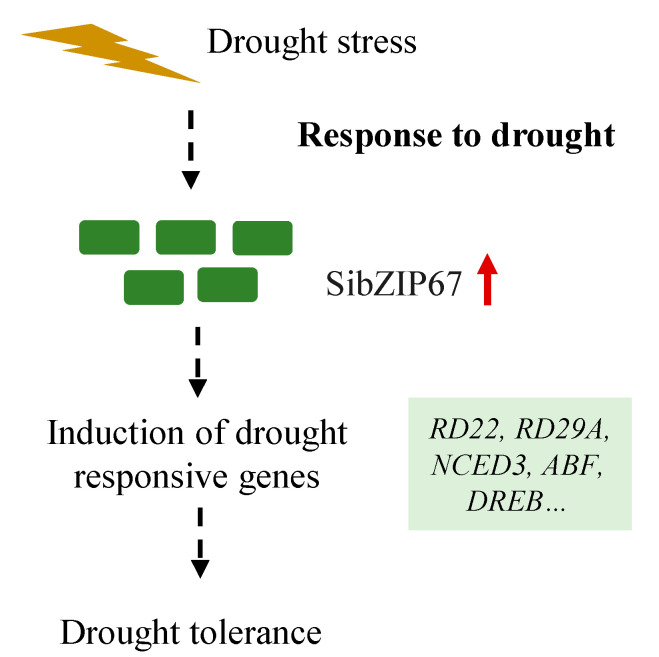
Proposed model of the positive roles of *SibZIP67* in drought tolerance.

The enzymatic antioxidant system plays an indispensable role in the early response of plants to drought stress [46]. Overexpressing *SibZIP67* in plants displayed reduced MDA accumulation and enhanced activities of antioxidant enzymes under drought stress (Figure 6). Previous studies have revealed that the role of ABA is the main plant hormone in the early responses to water stress [47]. To withstand drought, the plant reduces stomatal conductance to avoid losing water, and ABA plays a key role in this process [49]. The stress-responsive genes, *9-cis-epoxycarotenoid dioxygenase 3* (*NCED3*), *abscisic acid insensitive 1* (*ABI1*), *ABI5*, *ABF3*, *responsive to desiccation 22* (*RD22*), and *RD29,* are associated to ABA biosynthesis, signaling, and responding pathway [50,51]. These genes were also regulated after drought stress in *SibZIP67* transgenic plants in the study (Figure 7). These data indicated that the drought-induced expression level of *SibZIP67* regulates the expression of stress-related genes, thus improving plant drought tolerance (Figure 8). Studies have elucidated the mechanism by which the gene *OsbZIP66* enhances drought resilience [25,52]. OsbZIP66 together interacted with a coactivator phosphatidylethanolamine-binding protein, MOTHER OF FT AND TFL1 (OsMFT1), forming the OsbZIP66-OsMFT1 complex [25]. The OsbZIP66-OsMFT1 complex regulated the transcription level of rice *late embryogenesis abundant group 3* gene (*OsLEA3*), thereby promoting drought tolerance [25,52]. In monocots, *bZIP* genes also play critical roles in the drought stress of dicots [53]. In *Arabidopsis*, the well-known *bZIP* genes, like *ABA-responsive element 1* (*AREB1*), *AREB2*, and *ABRE-binding factor 3* (*ABF3*), are core components in the ABA-dependent pathway cooperatively regulating drought responses [53]. Given the pivotal role of *bZIP67* genes in our study and their differential responses in a drought-resistant millet variety compared to a sensitive one, as observed in prior studies [32,33], it would be profoundly insightful to delve into the intricate molecular regulatory mechanisms of *bZIP* genes in response to abiotic stresses. This exploration could potentially unveil novel strategies for enhancing crop resilience against environmental adversities.

## 5. Conclusions

A total of 92 *bZIP* genes were identified from the foxtail millet genome, and they were clustered into ten subfamilies using the phylogenetic tree. Differentially expressed genes in drought stress responses were identified using transcriptome data analysis, and four *SibZIP* genes were included. The expression of gene *SibZIP67* was verified using qRT-PCR analysis, and the gene was cloned and overexpressed in Arabidopsis. Overexpressing *SibZIP67* in *Arabidopsis* regulated key genes related to drought stress responses and improved plant drought tolerance. *SibZIP67* plays a positive role in the drought responses of foxtail millet.

## Data Availability

The data involved in this study are listed in the article and its Supplementary files.

## Supplementary Materials

The following supporting information can be downloaded at https://www.mdpi.com/article/10.3390/biom14080958/s1, Figure S1. The expression level of *SibZIP67* in transgenic lines and WT. Figure S2. Phenotype and germination rate of WT and *SibZIP67* overexpressing lines under ABA treatment; Table S1. Primers used in the study; Table S2. *SibZIP* transcription factor encoding genes; Table S3. The differential expression genes after PEG treatment in foxtail millet; Table S4. The expression level of *bZIP* genes after PEG treatment in foxtail millet.
