# Supplementary material for: Expression of Foxtail Millet bZIP Transcription Factor SibZIP67 Enhances Drought Tolerance in Arabidopsis
Source: Biomolecules. 2024 Aug 7;14(8):958. doi: 10.3390/biom14080958 (PMC11352937; doi:10.3390/biom14080958)
Supplement: Supplementary file 1 [file biomolecules-14-00958-s001.zip › Supplementary Materials.pdf]

Supplementary Materials

Figure S1. The expression level of *SibZIP67* in transgenic lines and WT.

Figure S2. Phenotype and germination rate of wild type and *SibZIP67* overexpression lines under ABA treatment.

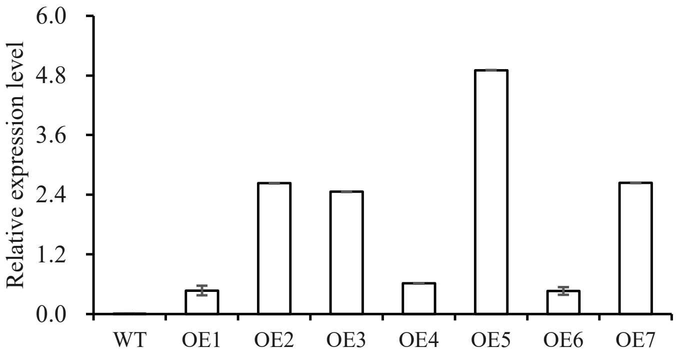

Figure S1. The expression level of *SibZIP67* in transgenic lines and WT. Each experiment contains three biological replicates, and each with two technical replicates (means of n = 6 ± SD).

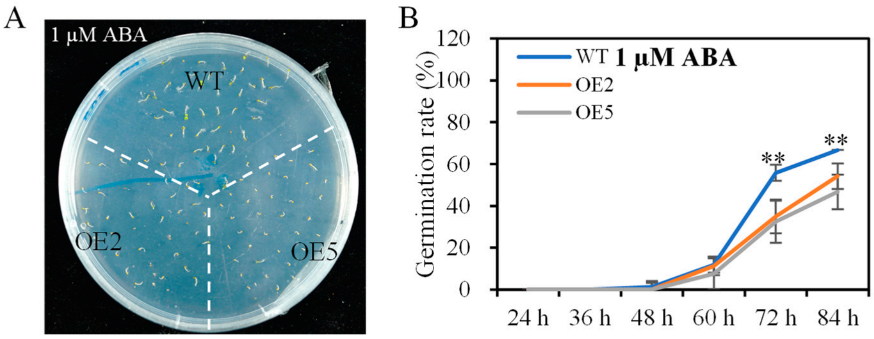

Figure S2. Phenotype and germination rate of wild type and *SibZIP67* overexpression lines under ABA treatment. (A) Phenotype of wild type and *SibZIP67* overexpression lines on 1/2 MS medium containing 1 μM ABA for 6 days. (B) Seed germination rates of wild type and *SibZIP67* overexpression lines on 1/2 MS medium containing 1 μM ABA. Three replicates were performed. Error bars indicate SD. * indicates significant difference ($P < 0.05$).
